# Efficiency of expanded noninvasive prenatal testing in the detection of fetal subchromosomal microdeletion and microduplication in a cohort of 31,256 single pregnancies

**DOI:** 10.1038/s41598-022-24337-9

**Published:** 2022-11-17

**Authors:** Huili Xue, Aili Yu, Min Lin, Xuemei Chen, Qun Guo, Liangpu Xu, Hailong Huang

**Affiliations:** 1 Medical Genetic Diagnosis and Therapy Center, Fujian Key Laboratory for Prenatal Diagnosis and Birth Defect, Fujian Maternity and Child Health Hospital College of Clinical Medicine for Obstetrics & Gynecology and Pediatrics, Fujian Medical University, No. 18 Daoshan Road, Gulou District, Fuzhou City, 350001 Fujian Province China; 2 Reproductive Medicine Center, Fujian Maternity and Child Health Hospital College of Clinical Medicine for Obstetrics & Gynecology and Pediatrics, Fujian Medical University, No. 18 Daoshan Road, Gulou District, Fuzhou City, 350001 Fujian Province China

**Keywords:** Molecular biology, Biomarkers, Health care, Medical research, Molecular medicine

## Abstract

Noninvasive prenatal testing (NIPT) is widely used to screen for common fetal chromosomal aneuploidies. However, the ability of NIPT-Plus to detect copy number variation (CNV) is debatable. Accordingly, we assessed the efficiency of NIPT-Plus to detect clinically significant fetal CNV. We performed a prospective analysis of 31,260 singleton pregnancies, included from June 2017 to December 2020. Cell-free fetal DNA was directly sequenced using the semiconductor sequencing platform for women with high-risk CNV with clinically significant results. Fetal karyotyping and chromosomal microarray analysis (or next-generation sequencing) are recommended for invasive diagnostic procedures. Women at low risk with no other abnormal results continued their pregnancies. We analyzed the expanded NIPT results, diagnostic test results, and follow-up information to evaluate its performance in detecting fetal CNV. Of the 31,260 pregnant women who received NIPT-Plus, 31,256 cases were tested successfully, a high risk of clinically significant CNV was detected in 221 cases (0.71%); 18 women refused further diagnosis; 203 women underwent invasive prenatal diagnosis; and 78 true positive cases and 125 false positive cases, with an overall positive predictive value (PPV) of 38.42% and a false positive rate of 0.40%. For known microdeletion/microduplication syndromes (n = 27), the PPVs were 75% DiGeorge syndrome (DGS), 80% 22q11.22 microduplication, 50% Prader–Willi syndrome, and 50% cri-du-chat. For the remaining clinically significant fetal CNVs (n = 175), the combined PPVs were 46.5% (CNVs > 10 Mb) and 28.57% (CNVs ≤ 10 Mb). NIPT-Plus screening for CNV has certain clinical value. NIPT-Plus yielded relatively high PPVs for 22q11.2 microduplication syndrome and DGS, and low to moderate PPVs for other CNVs.

## Introduction

Chromosomal anomaly, including submicroscopic copy number variation (CNV), is a major cause of birth defects^1^. Studies have shown that the proportion of fetuses carrying pathogenic CNVs can reach 1.6–1.7%, which is much higher than the prevalence of common fetal trisomies^2^. Noninvasive prenatal testing (NIPT) is more popular than the traditional maternal serum screening because of its higher sensitivity and specificity in screening trisomy 21, 18, and 13^3,4^, reducing unnecessary invasive diagnostic procedures and the associated risk of fetal loss^5^. With the rapid development of high-throughput sequencing and bioinformatics analysis, a growing number of studies have indicated that NIPT can be used to detect microdeletion/microduplication syndromes (MMSs)^6–8^.

A large proportion of CNVs can cause severe genomic diseases. Clinically relevant CNVs occur in 6% of fetuses with structural anomalies^9^. Unlike chromosomal aneuploidies, the risk of fetal CNVs is independent of maternal age^10^. Thus, it is beneficial to detect clinically significant fetal CNVs in all pregnant women, irrespective of maternal age, including younger pregnant women. Thus, the application of NIPT, expanded from common aneuploidies to MMS, will guide pregnancy management.

MMSs though relatively rare, collectively account for 1–2% of all congenital malformations in newborns^11^. Many commercially available NIPT cover the detection of specific MMS^12,13^. The NIPT-Plus showed varying performance in detecting specific MMS, with only low to moderate positive predictive value (PPV) of DiGeorge syndrome (DGS), 1p36 deletion syndrome, cri-du-chat (CDC), and Prader–Willi/Angelman syndromes (PWS/AS)^14–16^. In recent years, several studies have demonstrated that it is promising and feasible to utilize NIPT in detecting fetal MMS^10,17–27^. However, there have been few prospective large-scale population studies^8,19^, and its performance remains challenging and controversial.

At present, karyotyping and chromosomal microarray analysis (CMA) or CNV sequencing (CNV-seq), have been recommended to prenatally identify fetal clinically significant CNVs as a first-tier technique^28^. In this study, we prospectively investigated 31,256 singleton pregnancies using NIPT-Plus and investigated its efficacy in detecting fetal clinically significant CNVs.

## Material and methods

### Study subjects and expanded NIPT data sources

This prospective study enrolled 31,260 pregnant women with a singleton pregnancy who underwent NIPT-Plus, in Fujian Maternity and Child Health Hospital, of whom four failed to be detected. The study was approved by the Ethics Committee of the Hospital (2015KYLLD01051). All methods were performed in accordance with the relevant guidelines and regulations. The test method, screening-covered diseases, and limitations and risks were informed.

Blood samples were sent for the NIPT-Plus test, generating 36-bp genomic sequence reads. Reads were assigned to consecutive non-overlapping 100 Kb bins to further filter bins with low coverage and GC content < 30% or > 70%. Thus, data regarding data regarding clinically significant CNV cases were obtained from Ion Proton semiconductor sequencing platform (Da An Gene Co., Ltd., Shenzhen, China), the sequencing depth was 0.4 ×, and the data volume was 8 million reads. The ENET algorithm was applied to calculate the fetal fraction (FF)^29^. The redraw rate was 0.49%. The median FF of the samples passing quality control was 11.2% (4.0–48.3%).

### Pregnant woman demographics

**Figure 1:**
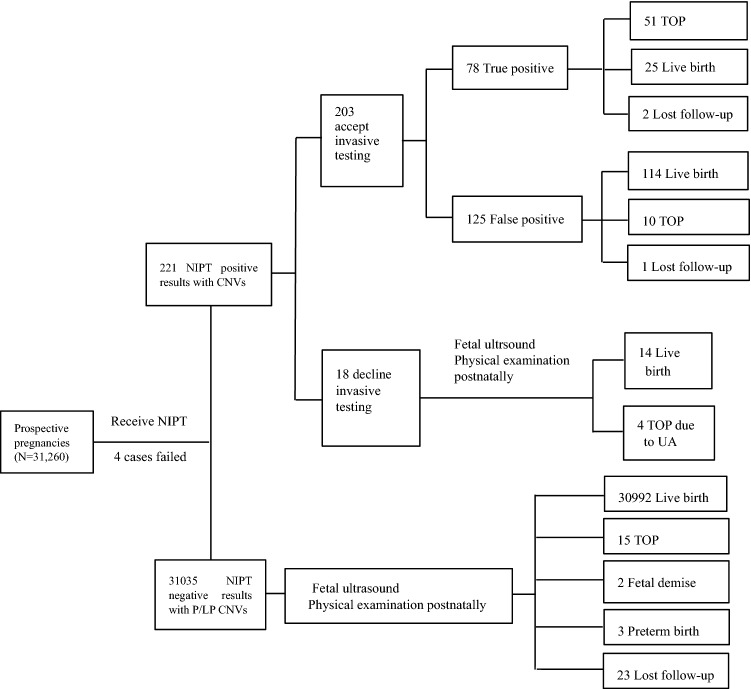
Flowchart of NIPT-Plus results and clinical outcome of pregnant women. *NIPT* noninvasive prenatal testing, *TOP* terminate of pregnancy, *UA* ultrasound anomaly. *CMA detection of induced labor tissue indicated true positive.

The demographic characteristics of maternal age, gestational week, gravidity, and prior screening are shown in Table 1. The pregnant women were 19–48 years old (mean age, 32.2 years). The range for gestational weeks at NIPT was 12–32 weeks. The mean values of gestational week at NIPT and invasive testing were 17.3 ± 2.0 and 22.7 ± 2.6 weeks, respectively. Prior maternal serological screening (MSS) tests before NIPT, including abnormal MSS results [high risk, critical risk and abnormal multiple of the median for single marker value (AFP, β-HCG, uE3)] and low risk. Invasive diagnositic procedures include amniocentesis during 16 and 24 gestational weeks, and fetal blood sampling beyond 24 gestational weeks. Autosomal and all chromosome aneuploidies were excluded. Totally, 221 women were suspected to have fetal CNVs, after genetic counseling, and 203 women voluntarily opted invasive testing by karyotype and CMA/CNV-seq, and 18 women refused invasive testing (Fig. 1).

**Table 1:** Clinical characteristic of pregnant women undergoing NIPT-Plus.

Variable	Value
**Age(years)**	**No. rate (%)**
19–26	6628 (15.3)
27–34	26,555 (61.3)
35–42	9877 (22.8)
> 42	260 (0.6)
**Gestational weeks**	
12–15^+6^	13,169 (30.4)
16–19^+6^	21,487 (49.6)
20–23^+6^	8231 (19.0)
24–26^+6^	390 (0.9)
≥ 27	43 (0.1)
**Specimens, n(%)**	
Amniotic fluid	100 (81.3)
Cord blood	23 (18.7)
**Pregnancy types**	
Singleton pregnancy	41,804(96.5)
Twin pregnancy	1516(3.5)
**Clinical features**	
AMA	10,137 (23.4)
Abnormal serologic screening(MSS)	12,953 (29.9)
High risk	2513 (5.8)
Critical risk	8794 (20.3)
Abnormal single marker MOM	1646 (3.8)
Only NIPT	17,371 (40.1)
Soft ultrasound markers	1733 (4.0)
Adverse reproductive history	390 (0.9)
Other*	736 (1.7)

*AMA* advanced maternal age, *NIPT* noninvasive prenatal testing, *n* number, *MoM* multiple of the median, *MSS* maternal serological screening.

*Pregnant women with contraindications for invasive diagnostic procedures.

### Invasive prenatal diagnostic testing by karyotyping and CNV analysis

For further validation, women with NIPT-positive clinically significant CNVs results were recommended to undergo invasive diagnostic procedures, and 30 mL of amniotic fluid or 4 mL of cord blood was obtained. In addition, in cases with NIPT-negative CNVs results, fetuses anomaly were examined by either ultrasound prenatally or physical examination postnatally, and were also advised chromosome testing.

Karyotypes were scanned on Leica GSL120. At least 20 metaphases were counted, and five metaphases were analyzed. The naming of abnormal karyotypes were based on ISCN 2020.

CMA was performed using Affymetrix CytoScan 750 K array (Affymetrix Inc., Santa Clara, CA), the experimental processes were performed as previously described^30^, and data analysis was carried out using Affymetrix Chromosome Analysis Suite Software (version 3.1.0.15). The reporting threshold was set at copy number gains/losses ≥ 500 Kb and loss of heterozygosity (LOH) ≥ 10 Mb.

In regard to CNV-seq, library construction and purification operation was conducted by Biosan chromosomal CNV assay kit (reversible terminal termination sequencing), the concentration of library was quantitatively determined by quantification through KAPA Library KTS. Post-quantitative library pooling was sequenced on Illumina NextSeq 500 sequencing platform at ~ 1 × depth, and software was used for chromosomal CNV above 0.1 Mb finally. The number of reads after sample quality control is more than 8 M Sequence depth is ~ 0.1 ×. Burrows-Wheeler algorithm for calculating CNV was performed according to the previous study^29^.

CNVs were classified through OMIM, UCSC, International Standard Cytogenomic Array, Database of Genome Variants, and Decipher databases into pathogenic, likely pathogenic, variants of unknown significance (VOUS), likely benign (LB), and benign. Data were analyzed using the human genome hg19 reference sequence. The pathogenicity significance of CNVs was evaluated following the ACMG guidelines^31^. For fetuses with confirmed abnormal CNVs, parental testing was performed to determine its origin.

NIPT result is true positive (TP) if confirmed by diagnostic testing of the fetus, mother, or placenta. When diagnostic testing of placenta, fetus, or mother do not confirm the NIPT results, the results are considered as false positive (FP), and when fetal chromosomal anomalies are detected which are not identified via NIPT, the NIPT results are considered false negative (FN). True negative (TN) refers to cases with negative NIPT results and the diagnostic test is normal.

### Follow-up and pregnancy management

All pregnant women received pre- and post-test counseling from a senior genetic counselor. Pregnant couples confirmed to have fetuses with pathogenic/likely pathogenic (P/LP) CNVs go through multi-disciplinary treatment, and they take an informed decision on whether to continue pregnancy. Follow-up began three months after delivery, including ultrasound examination report, diagnostic testing results, final pregnancy outcomes, infant’s sex, and physical examination of newborn results. Any FN clinically significant CNVs results subsequently identified by either ultrasound prenatally or physical examination postnatally were subjected to chromosome analysis. The pregnancy outcomes of the FN samples were recorded via telephone or through a follow-up registry.

### Statistics

SPSS software version 19.0 (SPSS, Inc., Chicago, IL) was used for statistical analysis. Measurement data were expressed as mean ± standard deviation, statistical comparisons were performed using χ^2^ test, and *p* < 0.05 was considered statistically significant.

### Ethics approval and consent to participate

This study was approved by the ethics committee of Fujian Maternity and Child Health Hospital, affiliated to Fujian Medical University (No. 2018KYLLD01051), and informed consent was obtained from all the pregnant women.

## Results

### Locations of CNVs detected by NIPT-plus

**Figure 2:**
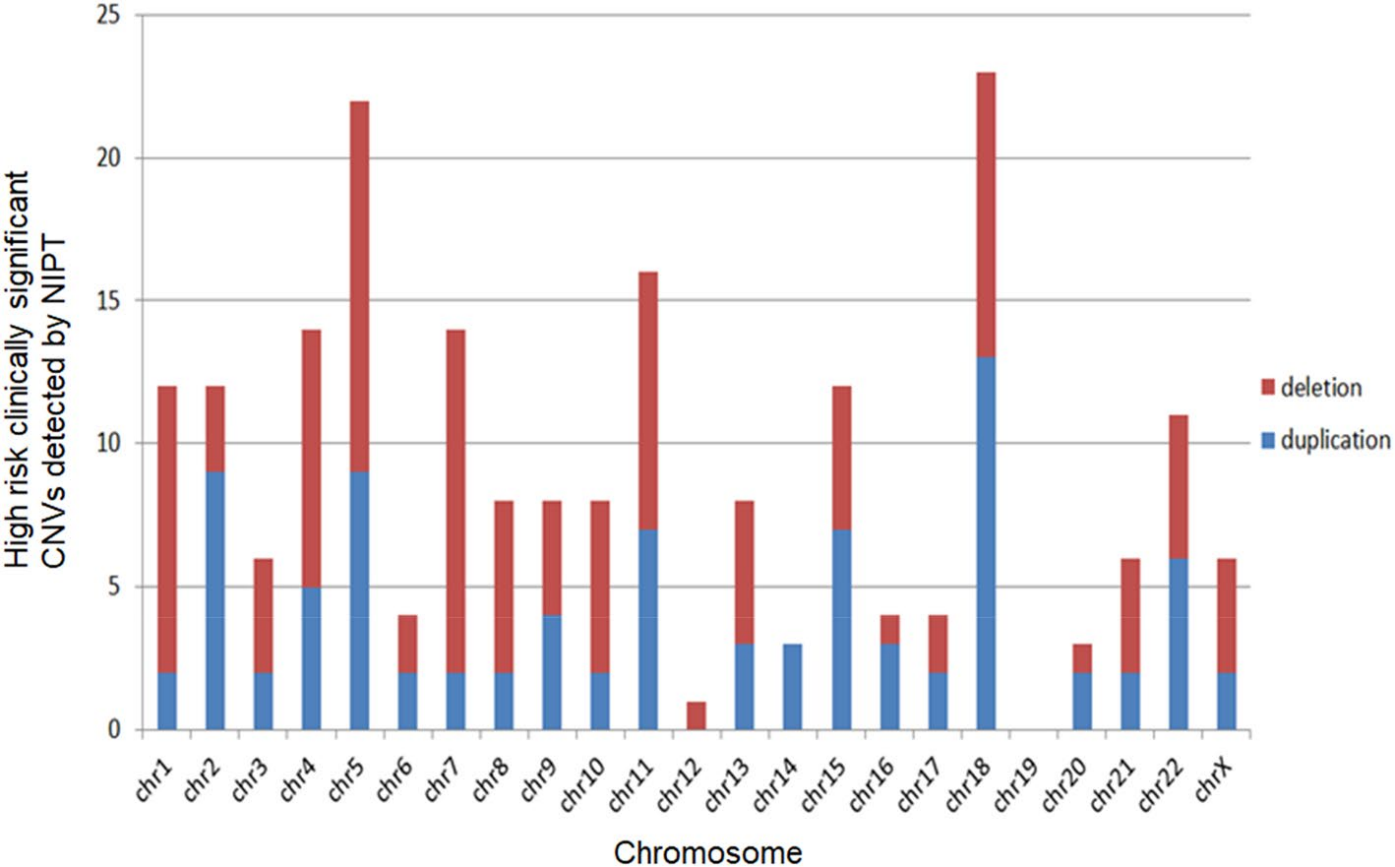
Autosomal and sexual CNVs detected by NIPT in 221 cases were distributed on each autosome and sexual except chromosome 19, and CNVs on chromosomes 4, 5, 7, 11, 15 and 18 were the most common. *CNV* copy number variation, *NIPT* noninvasive prenatal testing, *chr* chromosome.

Totally, 31,256 pregnant women who received NIPT-Plus were enrolled finally in this study due to 4 cases failed by NIPT-Plus. The enrollment, and flowchart are presented in Fig. 1. A total of 221 women were suspected to have fetal clinically significant CNVs, thus, the screening positive rate of fetal clinically significant CNVs was 0.71% (221/31,256), including 128 CNVs with microduplications ranging in size from 2.0 to 46 Mb and 98 CNVs with microdeletions ranging in size from 2.2 to 75.29 Mb (and more than one abnormality were detected in 5 cases), CNVs detected by NIPT were distributed in chromosome X and each autosome except chromosome 19, of which, and CNVs on chromosomes 4, 5, 7, 11, 15 and 18 were the most common, as shown in Fig. 2.

### Detection efficiency of NIPT-Plus in screening clinically significant fetal CNVs

Of the 221 cases with clinically significant CNVs, 203 (91.86%) cases underwent invasive diagnostic testing via amniocentesis or fetal blood sampling. The remaining 18 pregnant women decilined invasive testing. Among the 203 cases with clinically significant CNVs detected by invasive diagnostic testing, 78 cases were TP (of which, 33 were microduplications, 45 were microdeletions), and 125 cases were FP, with an overall PPV of 38.42% and an overall false positive rate (FPR) of 0.40%. Among 101 fetal positive confirmatory invasive diagnostic testing results, 59 were pathogenic, 11 were likely pathogenic, 25 were VOUS, 6 were LB (Table 2).

**Table 2:** A 101 fetal positive confirmatory invasive diagnostic testing results with fetal CNVs indicated by NIPT-Plus.

Case ID	MA	GW	NIPT-PLUS results	Fetal karyotype results	Fetal CMA/CNV-seq results/ pathogenicity classification	Fetal ultrasound finding	Pregnancy outcome	Chromosome disease syndrome indicated by NIPT-Plus
**Copy number gain TP (≤ 16 GW)**								
18	24	13^+6^	Dup 9q13 (~ 30 Mb)	46,XN,add(9)(?p13) pat	arr[GRCh37]9p13.1p24.3 (208,454_38,772,005) × 3 LP	FGR at 22 GW	TOP	Nonsyndromic
17	39	14	Dup 9p24.3p11.2 (~ 46 Mb)	46,XN,add(9)(?p24)	arr[GRCh37]9p24.3q21.13 (208,454_77,662,508) × 3 P	NT thickening	TOP	Nonsyndromic
27	37	14^+6^	Dup 13q31.1 (~ 4.3 Mb)	46,XN	arr[GRCh37]13q31.1 (78,894,976–81,994,976)) × 3 VOUS	None	Born (normal phenotype)	Nonsyndromic
7	23	15	Dup 17p12 (~ 3.34 Mb)	46,XN	arr[GRCh37]17p12 (14,060,293–15,416,912) × 3 P	None	Born (normal phenotype except high arch)	Charcot-Marie-Tooth 1A type syndrome
26	32	15^+6^	Dup 21q21.1q22 (~ 3.5 Mb)	46,XN	arr[GRCh37]21q21.3q22.11 (29,962,609_32,659,168) × 3 VOUS	None	Born (normal phenotype)	Nonsyndromic
6	32	16	Dup 2q23.3q24.2 (~ 9.93 Mb)	46,XN	arr[GRCh37]2q23.3q24.2 (154,042,539–162,258,705) × 3 dn LP	None	TOP	Non-syndromic
**TP (> 16 GW)**								
9	31	16^+3^	Dup 15q21.2 (~ 5.8 Mb)	46,XN	arr[GRCh37]15q11.2q13.1 (22,770,421_28,704,050) × 3 dn P	None	TOP	15q11-q13 duplication syndrome
25	26	17	Dup 4p13p12 (15 Mb)	47, XN, + mar	arr[hg19]4p13q12 (41,896,801–57,724,715) × 3 mat LP	None	TOP	Nonsyndromic
16	28	17^+1^	Dup 11q23.3q24 (~ 17 Mb)	46,XN	arr[GRCh37]11q23.3q25 (117,097,362_134,937,416) × 3 P	Dandy–Walker malformation	TOP	Nonsyndromic
5	29	17^+2^	Dup 22q11.21q12.1 (~ 2.0 Mb)	46,XN	arr[GRCh37]22q11.22q11.23 (22,997,928_25,043,045) × 3 mat P	None	Born (normal phenotype)	22q11 duplication syndrome
22	28	17^+3^	Dup 11q23.3q24 (~ 17 Mb)	46,XN	arr[GRCh37]11q23.3q25 (117,097,362–134,937,416) × 3 P	Dandy–Walker malformation at 23 GW	TOP	Nonsyndromic
21	26	18	Dup 15q11.21q13.1 (~ 6.3 Mb)	46,XN	arr[GRCh37]15q11.2q13.1 (22,102,621–28,315,618) × 3 pat* P	None	Born (normal phenotype)	15q11q13 duplication syndrome
19	38	18^+1^	Dup 3p26.3p22.3 (~ 35 Mb)	46,XN,add(3)(?p22)	arr[GRCh37]3p26.3p22.3 (1_43,682,691) × 3 pat P	HPE	TOP	Nonsyndromic
23	34	18^+5^	Dup 11q24.3q25 (~ 4.1 Mb)	46,XN	arr[GRCh37]11q24.3q25 (130,308,334–134,937,46) × 3 P	None	TOP	Nonsyndromic
10	36	19	Dup 5q14.2 (~ 10 Mb)	46,XN,add(5)(?p14)	arr[GRCh37]5q14.2 (90,230,935–91,382,020) × 3 LP	None	TOP	Nonsyndromic
4	35	19^+2^	Dup 22q11.2 (~ 2.7 Mb)	46,XN	arr[GRCh37]22q11.21 18,648,855–21,454,872) × 3 pat P	None	TOP	22q11 duplication syndrome
1	25	19^+2^	Dup 15q11.2 q13.1 (~ 4.9 Mb)	46,XN	arr[GRCh37]15q11.2q13.1 (23,281,885–28,526,905) × 3 mat P	None	TOP	15q11q13 duplication syndrome
28	29	19^+4^	Dup 17q21.3q31.32 (~ 3.0 Mb)	46,XN	arr[GRCh37]17q21.31 (44,187,491–44,784,639) × 3 mat VOUS	None	Born (normal phenotype)	Nonsyndromic
29	31	19^+5^	Dup 9q21.11q22.3 (~ 25 Mb)	46,XN	arr[GRCh37]9q21.11q22.31 (71,013,799–95,657,711) × 3 dn VOUS	None	Born (normal phenotype)	Nonsyndromic
15	25	19^+6^	Dup 4p16.315.2 (~ 25 Mb)	46,XN	arr[GRCh37]4p16.3p15.2 (68,345–25,296,039) × 3 P	None	TOP	Nonsyndromic
24	31	20	Dup 20q13.2q13.3 (12 Mb)	46,XN	arr[hg19]20q13.2q13.33 (51,504,974–62,913,645) × 3 P	None	TOP	Nonsyndromic
32	22	20	Dup13q12.11q12.13 (~ 3.0 Mb)	46,XN	arr[GRCh37]13q12.11q12.12 (22,073,046–25,230,759) × 3 LB	None	Born (normal phenotype)	Nonsyndromic
33	25	20	Dup 5p15.2p15.1 (~ 2.5 Mb)	46,XN	arr[GRCh37]5p15.2p15.1 (14,860,000–16,860,000) × 3 LB	None	Born (normal phenotype)	Nonsyndromic
3	20	20	Dup 22q11.21q11.21 (~ 2.88 Mb)	46,XN	arr[GRCh37]22q11.21 (18,648,855–21,461,017) × 3 mat P	None	TOP	22q11 duplication syndrome
2	24	20^+2^	Dup 22q11.2 (~ 4.0 Mb)	46,XN	arr[GRCh37]22q11.21 (18,919,477–21,915,207) × 3 mat P	None	Born (normal phenotype)	22q11 duplication syndrome
14	33	21	Dup 5p15.33p15.2 (~ 31 Mb)	46,XN,add(21)(p11.2)	arr[GRCh37]5p15.33p13.3 (113,584–32,448,253) × 3 P	None	TOP	Nonsyndromic
30	29	21	Dup 2p22.3 (~ 3.1 Mb)	46,XN	arr[GRCh37]2p22.3 (34,049,512–35,045,602) × 3 LB	None	Born (normal phenotype)	Nonsyndromic
11	31	21	Dup 5p15.33p15.2 (~ 15 Mb)	46,XN,add(21)(p11.2)	arr[GRCh37]5p15.33p13.3 (113,576–32,448,169) × 3 P	None	TOP	Nonsyndromic
8	33	21	Dup 2q23.324.2 (~ 9.9 Mb)	46,XN dup(2)(q23.3q24.2)	arr[GRCh37]2q23.3q24.2 154,042,539–162,258,705) × 3 dn LP	None	TOP	Nonsyndromic
13	31	23	Dup 20q13.2q13.3 (~ 12 Mb)	46,XN	arr[GRCh37]20q13.2q13.33 (51,504,974–62,913,645) × 3 P	Fetal lung cystic adenoma	TOP	Nonsyndromic
20	40	23^+1^	Dup 2q34q37.2 (~ 27.08 Mb)	46,XN,add(2)(?q34)	arr[GRCh37]2q34q37.2 (215,025,029–236,132,136) × 3 mat LP	Polyhydramnios	Lost follow-up	Nonsyndromic
12	39	23^+4^	Dup 5p15.33p11 (~ 41 Mb)	47,XN, + mar	arr[GRCh37]5p15.33p11 (113,576_46,242,541) × 3 P	None	TOP	Nonsyndromic
31	32	24	Dup 4p16.1 (~ 7.1 Mb)	46,XN	arr[GRCh37]4p16.2p15.33 (5,431,644–12,413,075) × 3 LB	None	Born (normal phenotype)	Nonsyndromic
**Copy number gain FP (≤ 16 GW)**								
35	35	14^+6^	Dup 6p25.3p22.2 (~ 4.2 Mb)	46,XN	arr[GRCh37]4p16.3 (68,345–4,277,002) × 1 dn P	None	TOP	Nonsyndromic
37	37	15^+6^	Dup 8p12 (~ 2.5 Mb)	46,XN	arr[GRCh37]16p11.2 (29,351,826–30,190,029) × 1 dn VOUS	Separation of renal pelvis	Born (normal phenotype)	Nonsyndromic
38	39	15^+6^	Dup 4p16.3 (~ 3.4 Mb)	46,XN	arr[GRCh37]5p13.2 (37,044,025–37,233,386) × 3 pat VOUS	None	Born (normal phenotype)	Nonsyndromic
**FP (> 16 GW)**								
39	43	16^+6^	Dup 7q21.11 (~ 5.1 Mb)	46,XN	arr[GRCh37]16p11.2 (29,428,531–30,176,508) × 1 pat VOUS	Separation of renal pelvis	Born (normal phenotype)	Nonsyndromic
34	27	17^+2^	Dup 2p12 (~ 2.6 Mb)	46,XN	arr[GRCh37]22q11.21 (18,301,185–21,184,093) × 3 P	Intracardiac echogenic focus	TOP	Nonsyndromic
36	34	17^+6^	Dup 2p12 (~ 2.1 Mb)	46,XN	arr[GRCh37]22q11.21 (78,345,501–79,629,001) × 3 dn P	None	TOP	Nonsyndromic
40	31	18^+1^	Dup 16p13.3p11.1 (~ 34.2 Mb)	46,XN	arr[GRCh37]16p13.12p11.2 (14628204–32924046) × 2 hmz VOUS	None	TOP	Nonsyndromic
**Copy number loss TP (≤ 16 GW)**								
78	36	14^+4^	Del 13q31.1 (~ 3.71 Mb)	46,XN	arr[GRCh37]13q31.1 (83, 494, 767–86, 543, 280) × 1 VOUS	None	Born (normal phenotype)	Nonsyndromic
69	38	14^+6^	Del 18p11.3p11.21 (~ 13 Mb)	47,XN, + mar	arr[GRCh37]18p11.32p11.21 (136,227–15,099,116) × 1 P	FGR, HPE	TOP	Nonsyndromic
50	32	15	Del 15q11q13 (~ 5.0 Mb)	46,XN	arr[GRCh37]15q11.2q13.1 (23,290,787–28,560,664) × 1 P	Intracardiac echogenic focus	TOP	PWS/AS
67	23	15	Del Xq22.3 (~ 45 Mb)	46,XN	arr[GRCh37]Xq22.3q28 (107,912,179–155,233,098) × 1 P	None	TOP	Nonsyndromic
45	25	15	Del 22q11 (~ 3.0 Mb)	46,XN	arr[hg19]22q11.21 (18,648,855–21,800,471) × 1 P	None	TOP	DGS
74	34	15	Del 4q31.3q32.2 (11 Mb)	46,XN	seq[hg19]4q32.1-q32.2 (155,800,001–164,960,000) × 1 LP	None	TOP	Nonsyndromic
72	23	15^+5^	Del 6q25 (~ 17 Mb)	46,XN	arr[GRCh37]6q25.1q27 (152,176,966–170,914,297) × 1 P	None	TOP	Nonsyndromic
**TP (> 16 GW)**								
70	30	16	Del 7q32q36 (~ 2.3 Mb)	46,XN	arr[GRCh]7q36.1 (149,828,703–152,102,066) × 1 P	None	TOP	Nonsyndromic
53	23	16^+2^	Del Xq23q28 (~ 3.68 Mb)	46,XX	arr[GRCh37]Xq28 (147550751–155233098) × 1 (female) P	Intestinal dilatation	Born (normal phenotype)	Nonsyndromic
68	30	16^+3^	Del 7q34q36 (~ 17 Mb)	46,XN	arr[GRCh37]7q34q36.38 (142,044,268–159,119,707) × 1 P	None	TOP	Nonsyndromic
42	37	16^+3^	Del 22q11.21 (~ 2.99 Mb)	46,XN	arr[GRCh37]22q11.21 (18,066,280–21,630,621) × 1 P	None	TOP	DGS
58	27	16^+4^	Del 18p11.32 (~ 3.0 Mb)	46,XN	arr[GRCh37]18p11.32p11.31 (2,186,353–5,675,587) × 1 mat P	None	TOP	Nonsyndromic
84	25	17	Del 11q22.3 (~ 5.18 Mb)	46,XN	arr[GRCh37]11q22.3 (104,181,493–106,629,690) × 1 mat LB	None	Born (normal phenotype)	Nonsyndromic
85^$^	33	17	Del 21q22.12q22.3 (~ 11 Mb)	46,XN	arr[GRCh37]21q22.12q22.3 ((36,746,514–48,093,361) × 1 P 8q24.22q24.3 (134,400,222–146,295,771) × 3 LP	None	TOP	Nonsyndromic
44	27	17	Del 22q11.21 (~ 2.8 Mb)	46,XN	arr[hg19]22q11.21 (18,916,842–21,800,471) × 1 P	VSD	TOP	DGS
66	25	17	Del 6q25 (~ 17 Mb)	46,XN	arr[GRCh37]6q25.1q27 (152,176,966–170,914,297) × 1 P	Intestinal dilatation	Lost follow-up	Nonsyndromic
60	28	17	Del 8q12.1q21.2 (~ 6.69 Mb)	46,XN	arr[GRCh37]8q12.3q13.2 (63,249,055–69,695,857) × 1 dn LP	None	TOP	Nonsyndromic
63^$^	28	17	Del 18q21.31q23 (~ 35 Mb)	46,XN,-18, + mar	arr[GRCh37]18p11.32p11.31 (136,227–3,348,254) × 1, P 18p11.31p11.21 (3,350,736–13,083,388) × 3, P 18p11.21 (13,090,666–15,170,636) × 1, P 18p11.21q21.31 (15,181,207–54,008,143) × 3, P 18q21.31q23 (54,020,488–78,013,728) × 1 P	Fetal aorta coarctation, pulmonary artery stenosis, VSD	TOP	Nonsyndromic
75	31	17^+2^	Del 1p31 (~ 4.5 Mb)	46,XN	arr[GRCh37]1p31.1 (78,282,099–84,553,373) × 1 VOUS	None	Born (normal phenotype)	Nonsyndromic
76	28	17^+2^	Del 18q22.3 (~ 2.2 Mb)	46,XN	arr[GRCh37]18q22.3 (69,288,001–71,535,501) × 1 VOUS	None	Born (normal phenotype)	Nonsyndromic
71	28	17^+3^	Del 9p24.3p24.2 (~ 2.5 Mb)	46,XN	arr[GRCh37]9p24..3p24.2 (208,454–2,920,085) × 1 P (152,093,040–159,118,443) × 2 hmz VOUS	Dandy–Walker malformation	TOP	Nonsyndromic
81	20	17^+6^	Del 15q25.2q26.3 (~ 18 Mb)	46,XN	arr[GRCh37]15q25.2q26.3 (83,759,214–102,397,317) × 1 VOUS	None	Born (normal phenotype)	Nonsyndromic
49	37	18	Del 15q11.2q13.1 (~ 5.0 Mb)	46,XN	arr[GRCh37]15q11.2q13.1 (23,290,787–28,659,911) × 1 P	None	TOP	PWS/AS
46	33	18^+1^	Del 22q11 (~ 2.5 Mb)	46,XN	arr[hg19]22q11.21 (18,636,749–21,136,749) × 1 P	None	TOP	DGS
52	33	18^+3^	Del 5p15.32p15.2 (~ 5.3 Mb)	46,XN	arr[GRCh37]5p15.33p15.2 (113,576–10,477,490) × 1 P	Single umbilical artery	TOP	Cri-Du-Chat
77	30	18^+6^	Del 15p13.1p14 (~ 2.7 Mb)	46,XN	arr[GRCh37]15q13.2q13.3 (30,241,910–32,991,173) × 1 VOUS	Subependymal cyst	Born (normal phenotype)	Nonsyndromic
73	28	19	Del 17p13.3–13.2 (4.2 Mb)	46,XN	arr[GRCh37]17p13.3p13.2 (525–4,669,796) × 1 P	None	TOP	Miller–Dieker syndrome
79	31	19	Del 11p15.1p13 (~ 11.18 Mb)	46,XN,del(11)(p13p15)	arr[GRCh37]11p15.1p13 (19,973,767–31,001,449) × 1 VOUS	None	Born (normal phenotype)	Nonsyndromic
59	26	19^+1^	Del 10q26.13q26.3 (~ 7.0 Mb)	46,XN,del(10)(q26.1)	arr[GRCh37]10q26.13q26.3 (125,262,198–135,426,386) × 1 P	None	TOP	Nonsyndromic
57	28	19^+2^	Del 17p13.213.3 (~ 4.2 Mb)	46,XN	arr[GRCh37]17p13.3p13.2 (525–4,669,796) × 1 P	None	TOP	Nonsyndromic
80	31	19^+2^	Del 4q26 (~ 10 Mb)	46,XN	seq[GRCh37]4q26 (114,340,000–119,800,000) × 1 VOUS	Enhanced liver echo	Born (normal phenotype)	Nonsyndromic
43	25	19^+2^	Del 22q11.21 (~ 3.72 Mb)	46,XN	arr[GRCh37]22q11.21 (18631365–21800471) × 1 P	None	TOP	DGS
65	27	19^+4^	Del 5q23.1 (~ 12 Mb)	46,XN	arr[GRCh37]5q21.3q23.1 (107,915,007–120,847,610) × 1 P	None	TOP	Nonsyndromic
47	32	19^+4^	Del 22q11.21 (~ 3.0 Mb)	46,XN	arr[hg19]22q11.21 (18,916,842–21,800,471) × 1 P	None	TOP	DGS
82	31	19^+5^	Del 9p23p13.1 (~ 24 Mb) Del 9q21.11q22.3 (~ 25 Mb)	46,XN	arr[GRCh37]9p23p13.1 (13,107,600–38,771,831) × 1 dn VOUS arr[GRCh37]9q21.11q22.31 (13,107,600–38,771,831) × 1 dn VOUS	None	Born (normal phenotype)	Nonsyndromic
83	31	19^+5^	Del 9p23p13.1 (~ 25.6 Mb)	46,XN	arr[GRCh37]9p23p13.1 (13,107,600–38,771,831)x1dn VOUS	Subependymal cyst	Born (normal phenotype)	Nonsyndromic
55*^$^	35	19^+5^	Del Xp22.31 (~ 2.6 Mb) Del 10q21.1 (~ 3.8 Mb)	46,XY	arr[GRCh37]Xp22.31 (6,455,152–8,141,076) × 0,mat (male) P 10q21.1 (55,657,551–57,504,582) × 1 VOUS	Fetal mild tricuspid regurgitation	Born (mild ichthyosis phenotype)	XLR ichthyosis
54	37	20	Del Xp22.31 (~ 21 Mb)	46,XY	arr[GRCh37]Xp22.31 (6455152_8135568) × 0 mat P	None	Born (mild ichthyosis phenotype)	XLR ichthyosis
62	33	21	Del 18q21.33q23 (~ 18 Mb)	46,XN,del(18)(?q21)	arr[GRCh37]18q21.33q23 (59,280,654–78,013,728) × 1 P	None	TOP	Nonsyndromic
48	30	22^+1^	Del 15q11.1q13.1 (~ 8.75 Mb)	46,XN	arr[GRCh37]15q11.2q13.1 (22,770,422–28,928,730) × 1 P	None	TOP	PWS/AS
61	34	22^+4^	Del 18q11.2q12.1 (~ 6.0 Mb)	46,XN	arr[GRCh37]18q11.2q12.1 (19,886,814–27,306,978) × 1 P	None	TOP	Nonsyndromic
51	31	22^+5^	Del 15q15.33p14.3 (~ 21 Mb)	46,XN,del(5)(p14.3)	arr[GRCh37]5p15.33p14.3 (113,577–21,810,739) × 1 P	None	TOP	Cri-Du-Chat
56	34	26	Del 4q31.3q32.2 (11 Mb)	46,XN	seq[GRCh37]4q32.1q32.3 (155,800,001–164,960,000) × 1 LP	Abnormal posture of right foot	TOP	Nonsyndromic
64	39	26^+2^	Del 13q32.1q34 (~ 17.8 Mb)	46,XN,r(13) (?p11q32) [61]/45,XX,-13^24^	arr[GRCh37]13q31.3q34 (94,929,201–115,107,733) × 1 P	FGR, absence of a-wave of ductus venosus, HPE, corpus callosum dysplasia	TOP	Nonsyndromic
41^$^	30	26	Del 18p11.3q22.3 (~ 5 Mb)	46,XN,r(18)(p11q22)[97]/46,XN,\idic r(18)(p11q22)^13^ /45,XN,-18^3^/47,XN,idic r(18) (p11q22) × 2^2^	arr[GRCh37]18p11.32p11.31 (136,227–3,334,683) × 1, LP 18p11.31q22.3 (3,342,699–72,722,952) × 3, P 18q22.3q23 (72,723,195–78,013,728) × 1 LP	None	TOP	Nonsyndromic
**Copy number loss FP (≤ 16 GW)**								
94	39	15	Del 18p23.3p23.1 (~ 5.1 Mb)	46,XN,15ph	arr[GRCh37]8p23.3p23.1 (168,483–6,999,220) × 2 hmz VOUS 8p23.1p12 (8,117,564–32,069,805) × 2 hmz VOUS	Atrial septal defect	Born (normal phenotype)	Nonsyndromic
90	34	15	Del 22q11.21 (~ 3.0 Mb)	46,XN	arr[hg19]4q13.2 (69,344,443–69,565,861) × 1 VOUS	None	Born (normal phenotype)	DGS
98	25	15	Del 1p36.32p36.31 (5.7 Mb)	46,XN	arr[GRCh37]4q24q25 (107033067–109404131) × 1 LP	None	TOP	1p36 deletion syndrome
95	29	15^+2^	Del Xp21.1q28 (~ 75.29 Mb)	46,XN	arr[GRCh37]8p21.2 (23,725,923–24, 936,161) × 3 pat VOUS	ARSA	Born (normal phenotype)	TS
88	25	15^+5^	Del 22q11.21 (~ 3.5 Mb)	46,XN	arr[GRCh37]4q13.2 (69,344,443–69,565,861) × 1 VOUS	None	Born (normal phenotype)	DGS
99	27	16	Del 7q22.1q31.1 (10 Mb)	46,XN	arr[GRCh37]11p14.1p12 (30,211,776–36,615,043) × 1 P	Bilateral pleural effusion	Born	Nonsyndromic
**FP (> 16 GW)**								
101	39	17	Del 15q11.2q13.1 (~ 4.2 Mb)	46,XN	arr[GRCh37]17p11.2 (16,727,490–20,433,723) × 1 P	VSD	Born	AS/PWS
89	26	17	Del 4q31-qter (~ 10 Mb)	46,XN	arr[GRCh37]4q32.3q35.2 (167230247–190921709) × 2 hmz VOUS	None	Born (normal phenotype)	Nonsyndromic
91	34	17^+2^	Del 2p13.3p11.2 (~ 4.9 Mb)	46,XN	arr[GRCh37]8q11.1q11.2 (46,919,156–51,932,566) × 1, VOUS 9p23(9,216,123–12,914,396) × 1 VOUS	None	Born (normal phenotype)	Nonsyndromic
93	34	17^+4^	Del 1q31.1q32.2 (~ 4.8 Mb)	46,XN	arr[GRCh37]6p22.3 (17,867,202–18,765,914) × 1 pat VOUS	None	Born (normal phenotype)	Nonsyndromic
86	28	18	Del 7q21.11q31.2 (~ 31 Mb)	46,XN	arr[GRCh37]22q11.21q11.22 (21,464,764–22,962,962) × 1 dn P	None	TOP	Nonsyndromic
87	25	18	Del 18p21.3q23 (~ 22.13 Mb)	46,XN	seq[hg19] dup (17)( p13.3p13.3) (1–712,489) × 3 LP	None	Born (normal phenotype except high arch)	Nonsyndromic
92	29	18^+6^	Del 21q22.3 (~ 3.4 Mb)	46,XN	arr[GRCh37]13q21.2 (59608821–60,709,021) × 1 pat VOUS	VSD	Born (normal phenotype)	Nonsyndromic
96	29	20	Del 13q12 (~ 3.2 Mb)	46,XN	arr[GRCh37]5q14.1 (76,983,283–77,512,158) × 3 mat LB	VSD	Born	Nonsyndromic
97	38	22	Dup Xq28 (~ 7 Mb)	46,XN	arr[GRCh37]4q31.3q32.2 (155,463,038–162,158,990) × 1 dn P	Complete endocardial cushion defect (unbalanced), coarctation of the aorta	Born	Nonsyndromic
100	35	23	Del 4p16.3p15.33 (12 Mb)	46,XN	arr[GRCh37]7q36.2q36.3 (152,747,657–159,119,707) × 1 P	FGR	Born (SGA)	WHS

*MA* maternal age, *GW* gestional weeks, LFU, *TOP* terminate of pregnancy, *MS-MLPA* methylation-specific multiplex ligation-dependent probe amplification, *mat* maternal, *pat* paternal, *P* pathogenic, *LP* likely pathogenic, *VOUS* variants of uncertain significance, *CMA* chromosomal microarray, *CNV* copy number variation, *NIPT* noninvasive prenatal testing, *TS* turner syndrome, *UA* ultrasound anomalies, *WHS* wolf-hirschhorn syndrome, *XLR* X-linked recessive, *SGA* small for gestational age, *ARSA* aberrant right abuclavian artery, *VSD* ventricular septal defect, *FGR* fetal growth restriction, *HPE* holoprosencephaly.

*Fetal MS-PLPA: methylation-specific multiplex ligation-dependent probe amplification, paternal duplication.

^$^When there are multiple CNVs, only the highest pathogenicity classification is calculated.

Among the 203 cases with validation, P/LP CNVs were identified in 70 cases, including 19 cases with susceptibility loci (SL) for neurodevelopmental disorders (NDD) and 18 cases with abnormal karyotypes. Of the 18 fetuses, 14 had confirmed CNVs ≥ 10 Mb and 4 had CNVs < 10 Mb. In addition, CMA also detected 5 LOH (Tables 2, 3).

**Table 3:** The overall PPV and the rate of TP in each of these two cohorts (at ≤ 16 weeks and > 16 weeks).

GW at NIPT	n	NIPT positive	TP	FP^$^	FN*	Refused invasive testing	PPV(%)
≤ 16	13,172	34	13	14	3	7	48.1
> 16	18,084	187	65	111	4	11	36.9

*^,$^13 of 18 pregnancies who declined invasive testing with no confirmed test and 23 pregnancies that have been lost follow-up with low-risk results were excluded when making data statistics.

There were 27 cases of CNVs associated with classical MMS. This comprised 8 cases at high risk of DGS, 5 cases at high risk of 22q11.2 microduplication syndrome, 6 cases of PWS/AS, 4 cases of CDC, and 4 cases of 1p36 deletion syndromes. Of the eight cases of suspected DGS, there were 6 TPs and 2 FPs yielding a PPV of 75%. Of the five cases of suspected 22q microduplication syndrome, there were four TPs and one FP, yielding a PPV of 80%. For the six suspected PWS/AS cases, there were three TPs and three FPs; for the four suspected CDC cases, there were two TPs and two FPs. Finally, four cases indicated as a 1p36 deletion proved to be FP (Table 4).

**Table 4:** Performance of NIPT-Plus for detection of clinically significant CNVs in 31,256 pregnancies.

Clinically significant CNVs	TP	FP/FPR	PPV	TN	FN/FNR	NPV	Specificity
**Classical MMS**	15	12/0.038%	55.56%	31,226	4/21.1%	99.99%	99.96%
22q11.2 deletion syndrome	6	2/0.006%	75%	31,247	1/14.29%	100%	99.99%
22q11.2 duplication syndrome	4	1/0.003%	80%	31,249	2/33.3%	99.99%	100%
Cri-du-Chat syndrome	2	2/0.006%	50%	31,252	0/0%	100%	99.99%
Prader–Willi syndrome/Angelman syndrome	3	3/0.01%	50%	31,250	0/0%	100%	99.99%
1p36 deletion syndrome	0	4/0.01%	0%	31,251	1/100%	100%	99.99%
**Other genome-wide CNVs**	63	113/0.36%	35.80%	31,077	3/4.55%	99.99%	99.64%
˃ 10 Mb	33	38/0.12%	46.5%	31,183	2/5.71%	99.99%	99.88%
≤ 10 Mb	30	75/0.24%	28.57%	31,150	1/3.23%	100%	99.76%
**Total**	78	125/0.40%	38.42%	31,046	7/8.24%	99.98%	99.60%

*CNV* copy number variation, *TP* true positive, *FP* false positive, *FPR* false positive rate, *PPV* positive predictive value, *TN* true negative, *FN* false negative, *FNR* false negative rate, *NPV* negative predictive value.

The remaining 175 cases of fetal CNVs were segmental CNVs that were classified as other genome-wide CNVs. Of these, there were 34 TPs and 40 FPs for CNVs ≥ 10 Mb (PPV, 45.95%) and 28 TPs and 73 FPs for CNVs < 10 Mb (PPV, 27.72%) (Tables 4, 5).

**Table 5:** The PPVs for all fetal CNVs indicated by NIPT-Plus according to different CNV sizes.

CNV size detected by NIPT	NIPT positive	TP	FP	Refused invasive testing	PPV(%)
Within 2–4 Mb	97	24	70	3	25.5
Within 4–7 Mb	29	14	13	2	51.9
Within 7–10 Mb	16	6	3	7	66.7
> 10 Mb	79	34	39	6	46.6
Total	221	78	125	18	38.4

*TP* true positive, *FP* false positive, *PPV* positive predictive value.

### Pregnancy outcomes

All pregnant women with TP NIPT results underwent genetic counseling to discuss pregnancy intention. While the majority of women diagnosed with fetal clinically significant CNVs elected termination of pregnancy (TOP), a relatively small proportion of pregnant women chose to continue their pregnancies. The TOP rates were much higher in pregnancies diagnosed with known MMS, including DGS (100%), PWS/AS (100%), and CDC (100%). In contrast, elective TOP rates were much lower in women carrying a fetus with 22q11.2 microduplication syndrome (50%) (Table 2).

Seven pregnancies with pathogenic CNVs were missed by NIPT-Plus (negative predictive value 99.98%) (Table 7). The FN cases with clinically significant CNVs included one of eight cases with confirmed DGS, one of five cases with confirmed 22q11.2 microduplication syndrome, one of four cases with confirmed 1p36 deletion syndrome, as well as a Wolf-Hirschhorn syndrome (WHS) case, a 8q24.22q24.3 duplication case, a 16p11.2 deletion case, and a 15q11.2 deletion case. In one of the seven (14.29%) FN cases, prenatal ultrasound detected fetal abnormalities, and pregnancies were terminated upon confirmation via invasive diagnostic testing. The remaining six FN cases were identified only at birth and subsequently confirmed by postnatal chromosome analysis. In the 3–12 months follow-up period after birth, no other FN cases were identified.

The underlying causes of these seven FNs were further investigated. In all seven pregnancies, low FF was unlikely because FF values ranged from 5 to 13.8%. The other five women refused further placenta studies; thus, each one case of WHS and 1p36 deletion syndrome was further investigated via placental tissue chromosomal analysis. From four placental biopsy samples, no evidence of 4p16.3 deletion and 1p36 deletion was identifiable, suggesting possible confined placental mosaicism (CPM) as a cause of the two FN results.

The follow-up of 18 pregnant women with high-risk CNVs detected by NIPT-Plus is shown in Table 6. Interestingly, there were three FP cases with normal fetal and placental anatomies but complicated with multiple 5–10.5 cm uterine leiomyomas detected via ultrasound, though their prior obstetrical and gynecologic history was negative. In one case, NIPT indicated a 38 Mb deletion at 7q21.11q31.31 (FF: 6.4%); in another case, NIPT indicated multiple CNVs involving chromosomes 3, 4, 7, 10, and 12 (a 40 Mb deletion at 3q25.2q29, a 20 Mb deletion at 4q24q28.1, a 70 Mb deletion at 7q11.23q34, a 24 Mb deletion at 10q22.3q24.31, and a 25 Mb deletion at 12q12q14.2 (FF: 10.8%). In the third case (case 86 in Table 2), NIPT indicated a 31 Mb deletion at 7q21.11q31.2; further CMA on amniocytes identified a 1.5 Mb microdeletion at 22q11.21q11.22, which is associated with DGS, and the pregnancy was terminated. Confirmatory CMA on amniocytes did not show any pathogenic CNV in the other two cases. CMA studies of the three placentas after induction or postpartum did not show the existence of abnormal CNVs.

**Table 6:** The follow-up of 18 pregnant women with high‐risk CNVs detected by NIPT-Plus refused invasive testing prenatally due to fetal ultrasound structural anomalies or contraindications to prenatal diagnosis.

Case ID	Prenatal ultrasound finding	Postnatal cord blood CMA results/pathogenicity classification)	Cord blood karyotyping	Associated disease with validation	Pregnancy outcome	NIPT-plus result
**1**	Complete endocardial cushion defect, hydramnios	Not done	Not done	No result	TOP	Del 20q11.23q13.31 (18 Mb)
**2**	FGR	arr[GRCh37]46, XY	46, XY	No result	Preterm birth at 35^+4^w, normal phenotype	Del 1p36.32p36.31 (5.5 Mb)
3	Bilateral pleural effusion	arr[GRCh37]3p26.3 (61,891–2,441,042) × 1 VOUS	Normal	No result	TOP	Del 7q22.1-q31.1 (8 Mb)
4	FGR,fetal BPD was less than the mean value 3.7SD, HC was less than the mean value 4.7SD	Not done	Not done	No result	TOP	Del 4p16.3p15.33 (13 Mb)
5	Fetal ventricular septal defect	Not done	Not done	No result	Born (normal phenotype)	Del Xp22.31 (~ 5.8 Mb)
**6**	Partial absence of corpus callosum	arr[GRCh37]46,XX	Normal	No result	Born (normal phenotype)	Del 1p36.32-p36.23 (~ 5.1 Mb)
7*	Fetal pelvic ectopic kidney with multiple cystic changes?	Not done	Not done	No result	Born (normal phenotype)	DupXq28 (~ 7 Mb)
**8**	Fetal FL and HL were less than the mean value 2SD	Not done	Not done	No result	Born (normal phenotype)	Del 10q25.310q26.3 (~ 16.8 Mb)
9	Right hydrocephalus	arr[GRCh37]46, XX	46, XX	No result	Born (normal phenotype)	Del 16p12.1-p11.2 (4.66 Mb)
10	Fetal double kidney echo enhanced	arr[GRCh37]46, XY	46, XY	No result	Born (normal phenotype)	Del 15q11.2–13 (5 Mb)
11	cerebellar dysplasia, smoon brain? strephenopodia	Not done	Not done	No result	TOP	Del 22q11.2 (5.2 Mb)
12*	Normal	Not done	Not done	No result	Born (normal phenotype)	Del 15q11.2–13 (4.5 Mb)
13	Normal	Not done	Not done	No result	Born (normal phenotype)	Del 4p16.3-p15.33 (12 Mb)
14*	Single umbilical artery	Not done	Not done	No result	Born (normal phenotype)	Del 7q22.1-q31.1 (10 Mb)
15	Intestinal echo enhancement	Not done	Not done	No result	Born (normal phenotype)	Del 10q25.3q26.3 (16.8 Mb)
16	Minimal pulmonary regurgitation, skin thickening of head and neck back	Not done	Not done	No result	Born (normal phenotype)	Dup 14q31.1q31.32 (22 Mb)
17*	Persistent left superior vena cava	Not done	Not done	No result	Born (normal phenotype)	Dup 2p25.3p24.3 (11.5 Mb)
18*	Fetal nasal bone dysplasia	Not done	Not done	No result	Born (normal phenotype)	Dup 4p16.17.1 (7.1 Mb)

*VOUS* variants of uncertain significance.

*Contraindications to prenatal diagnosis.

### Low‐risk NIPT CNV results

Of the 31,035 cases with fetal low-risk P/LP CNVs, 23 were lost to follow-up; thus, 99.93% (31,012/31,035) of thees cases were successfully followed. Twenty-five fetuses underwent diagnostic tests because of abnormal ultrasound findings. Of which, 24 cases showed normal karyotype as well as CMA results, and had normal live births. One fetus harbored pathogenic CNVs (FN-3 in Table 7), and the pregnant couple terminated the pregnancy given the test results. Among the 24 cases with normal karyotype and CMA results, one fetus died in utero owing to preeclampsia and multiple malformations, one fetus died in utero owing to oligohydramnios, and three fetuses were born preterm because of fetal growth restriction (FGR), premature rupture of membranes, intrauterine cytomegalovirus infection, respectively, and 15 women terminated the pregnancies due to fetal multiple ultrasound anomalies. No abnormalities were found in the remaining low-risk pregnant women during the 3–12 months postnatal follow-up.

**Table 7:** Seven cases with false negative NIPT results missed by NIPT-Plus with validation.

Case no.	Clinically significant CNVs by CMA	Z-score of CNV	Fetal fraction	Prenatal/postnatal findings
FN-1	arr[GRCh37] 22q11.21(18631365_21800471) × 1 DGS	− 2.51	9.1%	NOT identified by ultrasound prenatally, detected due to ventricular septal defect postnatally
FN-2	arr[GRCh37]1p36.33p36.32(849,466–4,894,800) × 1 1p36 deletion syndrome	1.34	11.58%	NOT identified by ultrasound prenatally, detected due to language retardation postnatally
FN-3	arr[GRCh37] 16p11.2(29,428,531–30,177,916) × 1 Nonsyndromic	2.78	5%	Fetal ultrasound anomalies: single umbilical artery, left renal parenchyma echogenicity enhancement, upper ureter dilatation on ultrasound
FN-4	arr[GRCh37] 4p16.3p15.2(68,345–22,489,538) × 1 WHS	− 2.97	13.8%	NOT identified by ultrasound prenatally, detected due to VSD postnatally
FN-5	arr[GRCh37]22q11.21 (18,919,477_21,915,207) × 3 22q11.2 duplication syndrome	2.56	10.8%	NOT identified by ultrasound prenatally, detected due to developmental delay and cleft palate postnatally
FN-6	arr[GRCh37] 8q24.11q24.3(117,830,985_146,295,771) × 3 Nonsyndromic	2.83	13.2%	NOT identified by ultrasound prenatally, detected due to seizure postnatally
FN-7	arr[GRCh37] 15q11.2(22,770,421–23,625,785) × 1 Nonsyndromic	2.71	6.8%	NOT identified by ultrasound prenatally, detected due to mental retardation postnatally

*DGS* DiGeorge syndrome, *WHS* Wolf-Hirschhorn syndrome, *FN* false negative, *VSD* ventricular septal defect.

## Discussion

Chromosomal abnormality is one of the most important causes of birth defects, and there is no effective method to deal with it. The aim of prenatal screening is to identify fetal chromosomal abnormalities. Currently, in comparison to traditional MSS for Down syndrome, NIPT screening for common trisomy and sex chromosome aneuploidy is more popular among pregnant women; however, it is still controversial whether NIPT should screen for MMS^2,32^. In the present study, we investigated the performance of NIPT-Plus for fetal P/LP CNVs in 31,256 pregnant women and assessed its clinical value.

Opponents have argued that the relatively low PPV, high FPR, and uncertain pathogenesis of CNVs cause a dilemma in interpreting reports on high-risk results, putting significant psychological stress on pregnant women, and even increasing unnecessary invasive diagnostic procedures and their associated risks. However, proponents have debated that the purpose of prenatal screening and prenatal diagnosis is to prevent the birth of infants with the burden of fetal chromosomal anomalies, even for MMS with low PPVs. Most MMSs occur randomly, because the risk of fetal CNVs is not related to the age of pregnant women, which is beneficial for pregnant women of all ages on NIPT screening for CNVs, which are observed in 1.0–2.0% of birth defects without ultrasound anomalies^33^. Individually, although the incidence of MMSs is low, they are more frequent than that of Down syndrome. Previous studies have shown that NIPT has a certain sensitivity in identifying some classic MMSs. In our study, 19 (9.36%, 19/203) fetuses harbored CNVs associated with SL for NDD; thus, NIPT would also provide the possibility of screening certain fetal chromosomal anomalies such as SL for NDD, which do not show significant abnormalities on ultrasound, such as neurodevelopmental abnormalities, mental retardation, developmental delays, autism, and so on, in addition, the size of CNVs associated with SL is usually below 5–10 Mb, which cannot usually be detected by karyotyping. Despite this, ACMG does not currently recommend NIPT screening for P/LP CNVs^31^. Recently, routine screening for MMS has been recommended for younger women because microdeletions are more common than aneuploidies^2^.

In this study, among 203 confirmed cases with fetal suscepted CNVs screened via NIPT, 78 were TP and 125 were FP, with an overall PPV of 38.42%, suggesting that NIPT demonstrates some efficiency in screening P/LP CNV, although the chance of FP cases was relatively high. In our study, P/LP CNVs through diagnostic testing were observed in 70 cases; Eighteen cases had abnormal karyotypes, of which fourteen fetuses with CNVs > 10 Mb and four fetuses with CNVs ≤ 10 Mb were confirmed (Table 2). Although NIPT has a relatively high FPR for CNVs, it is difficult to detect CNVs less than 10 Mb by conventional karyotype analysis. From this point of view, NIPT can compensate for the deficiency of karyotype analysis to some extent. Our data suggest that NIPT screening performance for CNV is not relatively good, which may be related to the small sample size, refusal of further diagnostic testing of a small proportion of pregnant women, CPM, and maternal CNV^34^. Overall, our data indicate that NIPT has clinical significance for the detection of fetal MMSs, which can provide an important basis for interventional prenatal diagnosis.

The study reported by Liang et al.^19^ showed that NIPT exhibited high sensitivity and specificity for the detection of clinically significant CNVs. In our study, for classic MMSs (n = 27), the PPV were 75% (DGS), 80% (22q11.22 microduplication), 50% (PWS/AS), and 50% (CDC). For the remaining clinically significant fetal CNVs (n = 175), combined PPVs were 45.95% (CNVs ≥ 10 Mb) and 27.18% (CNVs < 10 Mb), which is slightly higher than that reported by Liang et al.^19^ The slight difference may be related to the different sample sizes and NIPT sequencing depth.

For the classic MMSs in our study, the PPV for 22q11.2 microduplication syndrome in this study was very high (80%). The overall PPV for the detection of other MMSs varied. For DGS, PWS/AS, CDC, and 1p36 deletion syndrome, the PPVs were 75%, 50%, 50%, and 0%, respectively. The PPVs for PWS and CDC were slightly lower than those reported, with reported PPVs of 75% and 40%, respectively^19^. Petersen et al. reported that the PPV for CDC, PWS, 1p36 deletion syndrome, and DGS was 0%, 0%, 14%, and 21%, respectively^35^. Low-level CPM resulted in one FP case of DGS^36^; Thus, we speculate that CPM may also be the potential etiology in two FP DGS cases, three FP PWS/AS cases, two FP CDC cases, and one four 1p36 deletion cases.

The combined frequency of FN in MMS was 0.022% (7/31,256). These included one fetus identified via ultrasound prenatally and six detected only at birth. Thus, the frequency of FNs can be reduced to 0.019% by prenatal ultrasound examination. Thus, prenatal ultrasound results should be combined to consider the need for further invasive testing, consequently improving the detection rate of fetal MMSs^37^. We speculate that the FNs may be caused by biological factors other than a low FF. In two FN cases of WHS and 1p36 deletion syndrome, placental chromosomal analysis revealed no 4p16.3 and 1p36 deletion, which could explain the two FN NIPT results, suggesting possible CPM is considered as a cause of the two FN results. Although placental chromosome studies of the other four FNs cases are lacking, we speculated that low-level CPM might be the underlying cause of FN.

FP CNVs detected by NIPT can also be attributed to CPM^38^ and the death of a twin in utero. In this study, 125 of the 203 cases were confirmed to be FP. Unfortunately, placental biopsies were obtained after delivery or pregnancy termination for 15 of the 125 fetuses with normal genetic results, and 2 of them ultimately turned out to be CPM with CNVs, which presented with FGR. This supports the fact that CPM involving some P/LP CNVs may be associated with adverse pregnancy outcomes^39^. In our cohort, there were three FP pregnancies with normal fetal and placental anatomies but complicated with multiple 5–10.5 cm uterine leiomyomas detected via ultrasound. Further diagnostic results revealed that fetal and placental lesions were normal except in case 86. Thus, we speculated that uterine leiomyoma may confound the results of NIPT screening for CNV and lead to FP^40^. Therefore, when the medical history of the pregnant woman should be further understood when NIPT screening for CNV is positive.

Given the performance of NIPT for detecting MMSs in the present and other reported studies, compared to traditional serological screening, we propose that NIPT could be a candidate for first-line screening of pathigenic CNVs for all pregnancies, irrespective of maternal age^9^. Currently, there are no other methods available to screen for MMSs, although NIPT has a high FPR.

Factors that influence the performance of CNVs detection include CNV size, sequencing depth, FF, and GC content^41^. In our study, CNVs detected by NIPT were distributed in chromosome X and each autosome, except for chromosome 19. CNVs were frequently found on chromosomes 2, 4, 15, and 18. We speculated that chromosome 19 is very rarely involved, primarily because of its high GC content.

NIPT has a better detection performance for fetal CNVs ≥ 10 Mb at conventional sequencing depths^42^. However, their ability to detect smaller CNVs is reduced. In our study, the PPV of nonsyndromic CNVs greater than 10 Mb was slightly higher than that of CNVs less than 10 Mb detected by NIPT (45.95% vs 27.18%, *p* > 0.05), and our data showed that NIPT demonstrates good performance in detecting fetal CNVs, especially for CNVs ≥ 10 Mb, similar to the results of the study by Yu et al.^43^.

This study had some limitations. First, studies on placentas and maternal CNVs were not routinely conducted to explore the cause of discordance between NIPT results and normal invasive diagnostic results. Second, the sample size was not large enough, and further research is required to accumulate more data. Third, the data are based on a cohort from a single tertiary referral center, and there exists regional bias.

Fourth, the genetic information is incomplete due to 23 pregnancies with low-risk results that were lost follow-up and 18 pregnancies who declined invasive testing.

Our data indicate the potential significance of NIPT in screening clinically significant CNVs. NIPT exhibited high performance for the detection of 22q11.2 duplication syndrome and DGS, low to moderate detection performance for other clinically significant CNVs. We believe that NIPT-Plus combined with ultrasound examination and maternal history examination screening for MMS may be more effective in further multicenter studies with a larger population, increased sequencing depth, and improved bioinformatics analysis algorithms.

## Data Availability

The datasets used and/or analyzed during the current study are available from the corresponding author on reasonable request.

## Abbreviations

NIPT: Noninvasive prenatal testing
CNVs: Copy number variations
CMA: Chromosomal microarray analysis
LOH: Loss of heterozygosity
VOUS: Variants of unknown significance
AMA: Advanced maternal age
MMS: Microduplication/microdeletion syndrome
FPR: False positive rate
CPM: Confined placental mosaicism
NDD: Neurodevelopmental disorders
SL: Susceptibility loci
PPV: Positive predictive value
TP: True positive
FP: False positive
TN: True negative
FN: False negative
PWS/AS: Prader–Willi/Angelman syndromes
CDC: Cri-du-chat
DGS: DiGeorge syndrome
TOP: Termination of pregnancy
WHS: Wolf-Hirschhorn syndrome
